# Clinical evaluation of the loop-mediated isothermal amplification assay for the detection of common lower respiratory pathogens in patients with respiratory symptoms

**DOI:** 10.1097/MD.0000000000013660

**Published:** 2018-12-21

**Authors:** Jingyuan Hou, Heming Wu, Xing Zeng, Hui Rao, Pingsen Zhao

**Affiliations:** a Clinical Core Laboratory; b Center for Precision Medicine, Meizhou People's Hospital (Huangtang Hospital), Meizhou Academy of Medical Sciences, Meizhou Hospital Affiliated to Sun Yat-sen University; c Guangdong Provincial Engineering and Technologyl Research Center for Molecular Diagnostics of Cardiovascular Diseases; d Meizhou Municipal Engineering and Technology Research Center for Molecular Diagnostics of Cardiovascular Diseases; e Meizhou Municipal Engineering and Technology Research Center for Molecular Diagnostics of Major Genetic Disorders; f Guangdong Provincial Key Laboratory of Precision Medicine and Clinical Translational Research of Hakka Population, Meizhou 514031, P.R. China.

**Keywords:** bacterial culture, loop-mediated isothermal amplification, molecular diagnostics, pathogenic bacteria, respiratory tract infections

## Abstract

Lower respiratory tract infections (LRTIs) are a substantial public health problem and a leading cause of significant morbidity and mortality worldwide. The aim of this study was to evaluate a commercially available loop-mediated isothermal amplification (LAMP) assay for the simultaneously detection of thirteen common lower respiratory pathogens in patients with respiratory symptoms. All participants age from 1 to 101 years old were recruited from inpatient or outpatient of Meizhou People's Hospital between October 2016 and March 2018. A total of 1767 sputum samples and 88 bronchoalveolar lavage fluid samples from patients with suspected LRTI were collected. For each sample, a parallel study using both routine bacterial culture-based and LAMP assays were carried out. In total, 810 (44.85%) out of the 1855 samples were found to be positive infected with respiratory pathogens by using the LAMP assays. Methicillin-resistant *Staphylococcus aureus* (*MecA*) was the most predominant bacterial pathogens, with proportions of 17.09% in sputum and 10.23% bronchoalveolar lavage fluid samples, respectively. The proportions of bacterial pathogen infection with *Streptococcus pneumoniae (Spn)* (24.24%) was relatively high in aged <15 group (*P* <.001) while the proportions of bacterial pathogen infection with *MecA* (22.89%) was relatively high in aged >60 group (*P* <.001). Bacterial pathogen infection with *MecA* having the highest prevalence with proportions of 17.81% and 13.94% in male and female, respectively. A statistically higher proportion of male group had bacterial pathogen infection with *Pseudomonas aeruginosa* (*Pae*) in this study (*P* = .035). Comparison of results between the LAMP assay and culture method was conducted and our results indicated that there was higher detection rate by the LAMP assay than the bacterial culture method. Comparison of the results obtained with the LAMP assay and those obtained by sequencing analysis, when the sequencing method was set to 100%, demonstrating that the LAMP assay is 100% specific and 95.50% sensitive. The technique of LAMP assay was proved to be a simple, sensitive, specific, convenient, and rapid method, which can be implemented for diagnosing pathogenic bacteria in patients with LRTIs in primary labs without any need for expensive equipment or specialized techniques in resource-limited areas of China.

## 1 Introduction

Acute lower respiratory infections are a substantial public health problem and a leading cause of significant morbidity and mortality worldwide.^[1,2]^ Lower respiratory tract infections (LRTIs) is an umbrella term that encompass various aetiologies, which is associated with a large variety of different viral and bacterial pathogens. Owing to the complicated clinical manifestations of LRTIs, global efforts to reduce the burden of LRIs using different preventive and treatment strategies require timely information about the associated pathogens.^[3–5]^ Traditional diagnostics have been developed, such as smear microscopy, bacterial culture, biochemical tests, and serological tests.^[6–8]^ Nevertheless, these commonly used methods in clinical diagnosis were subject to several shortcomings, including tedious operation, special culture conditions, unaffordable time-consumption, insufficient sensitivity and specificity.^[9,10]^ Therefore, an urgent need for more rapid, sensitive and specific method in the field of early diagnosis of pathogen in LRTIs is still challenging.

Among molecular diagnostic methods, polymerase chain reaction (PCR) and real-time PCR assay are essential to quickly confirm a pathogen identification compared to culturing-based methods.^[11,12]^ Genetic testing using multiplex PCR is much faster and can also screen for multiple pathogens simultaneously.^[13,14]^ However, to perform PCR analysis, a molecular biology laboratory with highly sophisticated instrumentation, expensive regent and complicated operations is needed. Furthermore, the data obtained by PCR must be analyzed using complex computer software and interpreted by trained staff has impeded its usefulness.^[15,16]^ Recently, a novel means of nucleic acid amplification method termed the loop-mediated isothermal amplification (LAMP) was developed and can be an effective method to address deficiencies of PCR methods.^[17–19]^ The LAMP reaction employs a bacillus stearothermophilus DNA polymerase (Bst) with strand-displacement activity and a set of suitable primers that bind to 6 distinct regions on the target DNA, which makes the initial denaturation step obsolete and can be performed under isothermal conditions without costly equipment such as thermal cycler equipment.^[20–22]^ Based on these properties, the LAMP approaches have been widely used for the detection of pathogens in clinical diagnostics as a rapid, accurate and cost-effective method.^[23–26]^ Additionally, it was demonstrated that LAMP assay can be combined with microfluidic chips for improved practicability in clinical diagnostics and it can be appropriate in primary hospitals.

In the present study, our aim was to evaluate a commercially available respiratory pathogens nucleic acid thermostatically amplified kit regarding throughput, automation and analytical performances, and compared to traditional bacterial culture method.

## 2 Materials and methods

### 2.1 Patients

A cross sectional study was conducted at the Meizhou People's Hospital (Huangtang Hospital), Meizhou Hospital Affiliated to Sun Yat-sen University, Guangdong province, China. All participants age from 1 to 101 years old were recruited from inpatient or outpatient of Meizhou People's Hospital between October 2016 and March 2018. Patients who initially diagnosed as clinical suspicion of LRTIs were enrolled in this study if they met one of the following inclusion criteria:

(1) typical characteristics of pneumonia or bronchitis, which were firmly inferred from chest X-rays or computed tomography (CT) scan
(2) one or more respiratory symptoms, including exacerbated dyspnea, exacerbated sputum production, purulence or/and fever >38.5°C.

Exclusion criteria from enrolment were as follows: patients with non-infectious diseases, patients with infection by viruses or fungi. The ethical approval of the study protocol was obtained from the Human Ethics Committees of Meizhou People's Hospital (Huangtang Hospital), Meizhou Hospital Affiliated to Sun Yat-sen University, Guangdong province, China, and written informed consents were obtained from each participant.

### 2.2 Sample preparation and DNA extraction

The sputum or bronchoalveolar lavage fluid were collected into a sterile vial according to routine procedure. For each sample, a parallel study using both routine culture-based and LAMP assays were carried out. Briefly, the sputum specimen was transferred into to a sterile sealed container and subsequently decontaminated by adding an equal volume of 10% NaOH solution, vortex mixed for 5 minutes, follow by incubation at 37°C for 30 minutes. 1 mL of the supernatant derived from samples processed above or the bronchoalveolar lavage fluid was transferred into tubes and subsequent concentration by centrifugation at 12,000 rpm for 5 minutes, and then, the supernatant was discard and precipitate were used for DNA extraction. The extraction of genomic DNA of the bacterial pathogens was performed by using the Universal Kit for Bacterial DNA Extraction Kit (CapitalBio, Chengdu, China) following the manufacturer's recommended protocol. Quality and concentration of the purified DNA samples were measured and evaluated using Nanodrop 2000 TM Spectrophotometer (ThermoFisher Scientific, Waltham, MA) by examining OD260/OD280 and OD260/OD230. The isolated DNA was stored at −20°C until further use.

### 2.3 Pathogenic bacteria detection

**Figure 1:**
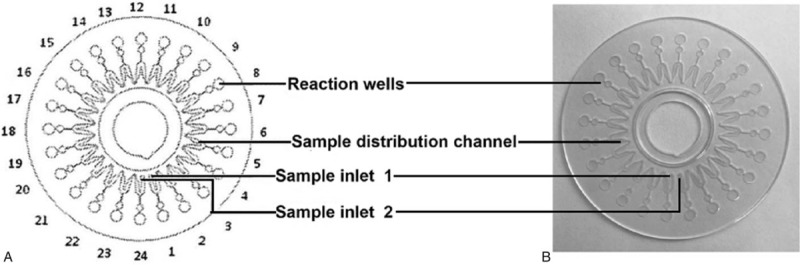
Schematic of the microfluidic chip consisting of an array of 24 reaction wells and microchannel for sample inlet, distribution and reaction. (A) schematic drawing of microfluidic chip and (B) photograph.

**Figure 2:**
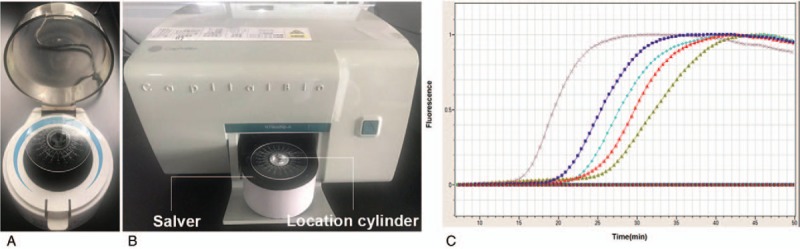
Microcentrifuge and setup CapitalBio RTisochip. (A) Microcentrifuge that used to make the mixture drop into the bottom of the reaction wells. (B) CapitalBio RTisochip-A equipped with a temperature-controlled system and a real-time imaging system. (C) Amplification curves for respiratory pathogens detection obtained using the microfluidic chip.

The LAMP detection of pathogenic bacteria was performed of Pathogenic Bacteria Nucleic Acid Detection Kit (CapitalBio Technology, Beijing, China) following the manufacturer's instructions based on a combination of isothermal amplification method and microfluidic chip method. The schematic and the amplification curves for respiratory pathogens detection obtained using the centrifugal force-driven microfluidic chip were presented as shown in Fig. 1 and Fig. 2, respectively. The limit of detection for the pathogenic bacteria is 500 copies per reaction. Briefly, 20 μL reaction regent and 34.5 μL DNA sample were mixed and then 50 μL of the mixture was simply added into microfluidic chip through the distribution channel due to pressure generated by the pipettor using a 200 μl pipette tip. In the process, air inside the chip escapes through air vents downstream of each reaction well, the inlet ports are covered with tape to prevent contamination. The chip is then placed in a microcentrifuge and follow by centrifugation at 3000 rpm for 30 seconds (Fig. 2A), then making the mixture drop into the bottom of the reaction wells so as to complete the detection course without cap opening. The reactions were performed on a RTisochipTM-A thermostatic expansion microfluidic chip nucleic acid analyzer (Fig. 2B), along with the real-time imaging system ((Fig. 2B)(CapitalBio Technology, Beijing, China), according to the following protocol: 1 cycles of 3 minutes at 37°C and 1 cycles of 47 minutes at 65°C. To visualise the results, respiratory tract pathogen nucleic acid detection software was used to analyze. The amplification and detection of the 13 bacterial pathogens were as follow: *Streptococcus pneumoniae* (*Spn*), *S.aureas* (*Sau*), *Methicillin-resistant staphylococcus aureus* (*MecA*), *Escherichia coli* (Eco), *Klebsiella pneumoniae* (*Kpn*), *Pseudomonas aeruginosa* (*Pae*), *Acinetobacter baumannii* (*Aba*), *Stenotrophomonas maltophilia* (*Sma*), *Haemophilus influenzae* (*Hin*), *Legionella pneumophila* (*Lpn*), *Mycoplasma pneumoniae* (*Mpn*), *Chlamydia pneumoniae* (*Cpn*), and *Mycobacterium tuberculosis complex* (*Mtb*). The results were discarded for any specimen with a negative internal control. Routine bacteria culture was performed independently in accordance with following respiratory pathogenic microorganisms operating standards: the samples were seeded on bacteriological media such blood agar plate, chocolate agar plates and blue agar plates using sterile wire loops and incubated at 35°C for 72 hours in a thermostatic incubator, and subsequently the dominant colonies were picked for bacterial detection using VITEK2 from BioMérieux (France) automatic bacterial analyzer. For those samples with discordant results, the samples were randomly selected and subjected to DNA sequencing for the presence of respiratory pathogens and the corresponding nucleotide sequences were used as criteria to identify sensitivity or specificity results.

### 2.4 Statistical analysis

SPSS statistical software version 19.0 was used for data analysis. All of the 1855 sputum and bronchoalveolar lavage fluid samples were simultaneously detected by the LAMP assay and bacteria cultures. The agreement between these 2 methods was evaluated by Kappa coefficient with a 95% CI for each pathogen. The comparison of the proportion for pathogen infection among genders and age groups was conducted using a Chi-square test or Fisher exact test, where appropriate. Statistical significance was defined as *P* <.05.

## 3 Results

Analyses were conducted on all evaluable patients (1353 males and 502 females) with available sputum or bronchoalveolar lavage fluid samples. Finally, a total of 1767 sputum samples and 88 bronchoalveolar lavage fluid samples from patients with suspected LRTI were collected. Baseline characteristics of the patients about the patients and samples were shown in Table 1.

**Table 1:**
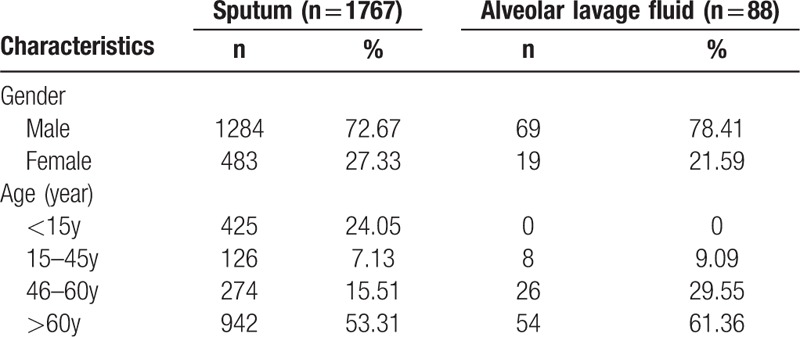
Baseline characteristics of the patients.

The identification and characteristic of the bacterial etiology was assessed descriptively as the number and percentage with positive results in both samples. There are thirteen different respiratory pathogens were identified in this study by the LAMP assay. In total, 810 (44.85%) out of the 1855 samples were found to be positive infected with respiratory pathogens by using the LAMP assay, as shown in Table 2. Among them, 554 samples (29.87%) were found to be single positive for bacterial pathogens infection, 160 samples (8.63%) were found to be double positive bacterial pathogens infection, and 96 samples (5.18%) were found to be multiple positive bacterial pathogens infection. In addition, *MecA* was the most predominant bacterial pathogens, with proportions of 17.09% in sputum and 10.23% bronchoalveolar lavage fluid samples, respectively. In general, our analysis has also shown that the positive detection rate of pathogenic bacteria in sputum samples was higher than that in bronchoalveolar lavage fluid samples.

**Table 2:**
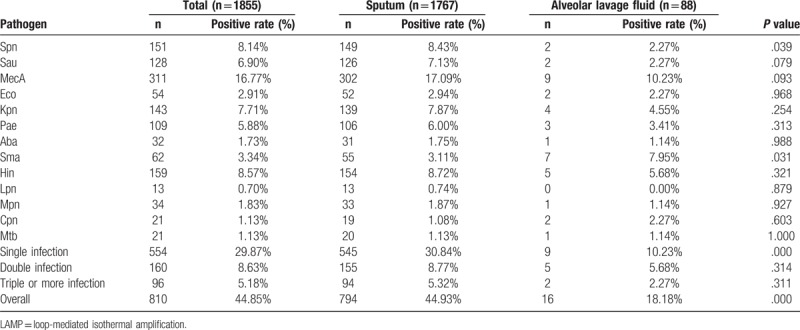
Distribution of pathogens detected by LAMP assay.

The relationship between pathogen and age were observed. As shown in Table 3, 1855 cases were divided into 4 groups according to the age (age <15, 15–45, 46–60, >60). Most bacterial pathogen infection occurred in patients aged <15, 46–60, >60, with corresponding proportions of 9.97%, 7.06%, and 24.85%. It is worth noting that the proportions of bacterial pathogen infection with *Spn* (24.24%) was relatively high in aged <15 group (*P* <.001) while the proportions of bacterial pathogen infection with *MecA* (22.89%) was relatively high in aged >60 group (*P* <.001). Multiple infection made up about 7.03% of the aged >60 group, other age groups include <15 (1.41%), 15 to 45 (4.48%) and 46 to 60 (4.67%). The relationship between pathogens and gender were also observed, as summarized in Table 4. Bacterial pathogen infection with *MecA* having the highest prevalence with proportions of 17.81% and 13.94% in male and female, respectively. A statistically higher proportion of male group had bacterial pathogen infection with *Pae* in this study (*P* = .035). There was a higher proportion of single infection of respiratory pathogen in the male group as compared to female group.

**Table 3:**
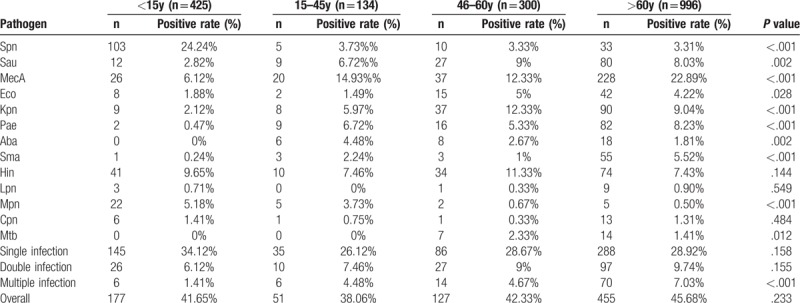
Relationship between pathogens and age.

**Table 4:**
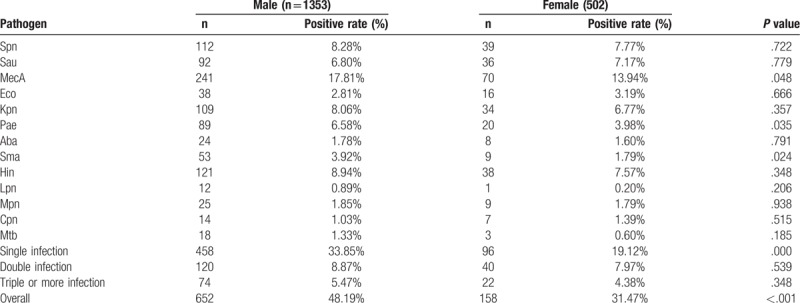
Relationship between pathogens and gender.

Comparison of results between the LAMP assay and culture method was conducted. The positive cases identified by LAMP assay and bacterial culture for each samples were summarized in Table 5. Out of the 1238 samples that were detection positive using the LAMP assay, 137 samples were positive while 1101 samples were negative by bacterial culture method, respectively. Meanwhile, of the negative samples detected using the LAMP assay, 124 samples were positive by bacterial culture method. The agreement between assays for each pathogenic bacterium was further analyzed through use of a Kappa coefficient with 95% CI. The bacterial with the largest agreement coefficient was *Pae* (0.311, 95% CI 0.982–0.991). Our results indicated that there was higher detection rate by the LAMP assay than the bacterial culture method.

**Table 5:**
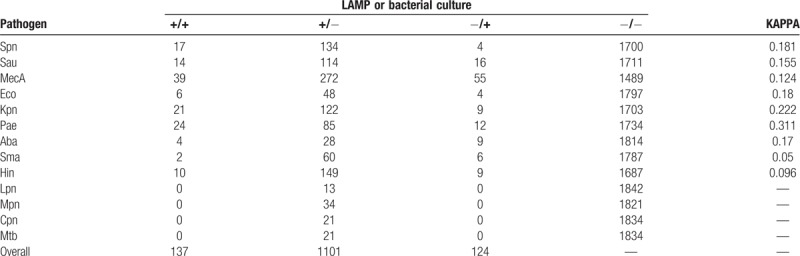
Comparison of the coincidence rate of pathogens detection between LAMP assay and traditional culture method.

To evaluate the diagnostic sensitivity and specificity of the LAMP assay, a total of 190 clinical respiratory samples were tested for the presence of pathogen by using the LAMP assay and DNA sequencing analysis. As summarized in Table 6, 85 and 101 samples were found to be positive and negative by the 2 methods. Overall, the sensitivity and specificity of the LAMP assay were 95.50% and 100%, respectively. These results implied that the LAMP assay has strong specificity and high sensitivity for detection of clinical samples.

**Table 6:**
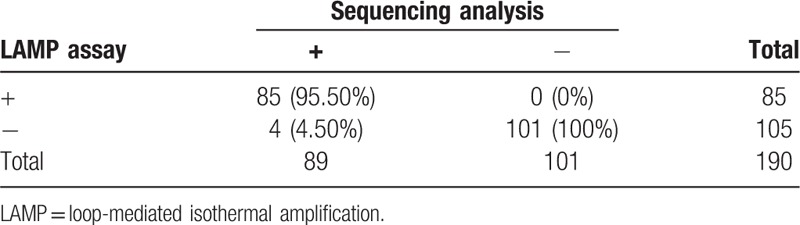
Sensitivity and specificity of the LAMP assay verified by sequencing analysis.

## 4 Discussion

LRTIs include severe diseases of the lower airways, such as acute bronchitis, bronchiolitis, and infections in patients with bronchiectasis pneumonia and is considered as the major causes of incidence and mortality over the past decades.^[1,2]^ Timely diagnosis of urgent life-threatening respiratory diseases is particularly important for optimal utilization of clinical therapy. Rapid and sensitive detection techniques, especially in patients suffering from LRTIs, are therefore in high demand.^[6,10,15]^ Molecular techniques such as LAMP are increasingly used in pathogens detection because of their rapidity, time efficiency, simple operation and cost-effectiveness, when compared to widely-used quantitative real-time PCR (qPCR) assays.^[27,28]^ In this study, our aim was to evaluate a commercial available LAMP assay for the simultaneously detection of thirteen common lower respiratory pathogens in patients with respiratory symptoms.

In this descriptive study, we first investigate the clinical characteristics and prevalence of LRTI among patients who were typical characteristics of pneumonia or bronchitis or with respiratory symptoms in a tertiary hospital setup in Meizhou region. LRTI caused by *MecA* was the largest groups in our population, with proportions of 17.81% and 13.94% in male and female, respectively. In addition, the proportions of bacterial pathogen infection with *MecA* (22.89%) was relatively high in aged >60 group (*P* <.001) As is widely known, bacterial pathogen infection with *MecA* is inundant in Chinese hospitals and in many other parts of the world, being responsible for a high burden of significant healthcare costs, morbidity, and mortality every year.^[29–31]^ The proportions of bacterial pathogen infection with *Spn* tested positive made up 5% among patients aged <15 group (*P* <.001). *Spn* is an encapsulated diplococcus causing a wide spectrum of diseases ranging from such as pneumonia, meningitis, and sepsis to noninvasive diseases like otitis media and sinusitis and even death.^[32]^ It is recognized that *Spn* infections to be one of the most frequently isolated bacterial pathogens and are the leading vaccine preventable causes of mortality in children under the age of 5 worldwide.^[33,34]^ Moreover, a total of 810 patients were tested positive for 1 or more type of respiratory bacterial pathogen. Of these, co-infections were found to be positive in 8% of the total recruited patients. The co-infections with multiple pathogens may have a worse effect on the progression of LRTI and better access to effective detection strategies is of the utmost importance and urged needed.

LRTI and the resulting potential presence of human enteric pathogens is a predominant threat to public health worldwide. Such a challenge and demand have provided a caveat for researchers to develop innovative techniques for respiratory bacterial pathogen detection using precision, rapidity, and user friendliness.^[11,20]^ However, the most extensively used detection methods at present for respiratory bacterial pathogen in developing countries are still the traditional smear microscopy and bacterial culture.^[7–9]^ In recent years, isothermal amplification methods have become a useful alternative to PCR, allowing molecular diagnostics due to their simplified setting and superiority in avoidance of aerosol contamination. Several LAMP-based protocols have been successfully developed and applied for the effective detection of pathogenic organism.^[22–24]^ In our study, the LAMP assay was performed for the detection of pathogenic bacteria in patients with LRTI, the bacteria amount excreted from lower respiratory tract was very little or absent, the detectable rate of both the sputum or bronchoalveolar lavage fluid samples was relatively too low to diagnose the LRTI by culture method. On the other side, the diagnostic method used in this study, which combined isothermal amplification methods and microfluidics chip, displaying high specificity and sensitivity. The discrepancy between the results of culture and genetic-screening methods is a recognized phenomenon which has been reported previously.^[35,36]^ LAMP based assays were highly consistent with clinical validation of these samples using sequencing analysis and the sensitivity and specificity found to be 95.5% and 100%. Microfluidic chips with a multitude of separate reaction wells, each containing primers for amplification of a specific pathogen, provide a promising platform for multiplexed detection and superiority in avoidance of aerosol contamination. Meanwhile, the Bst DNA polymerase used in LAMP assay is much less susceptible to inhibitory substances than other polymerases such as Taq,^[37]^ which enable the LAMP assay can generates much higher amounts of product DNA than PCR does, and the LAMP products can quickly be made visible to the real-time imaging system by the addition of a DNA-intercalating fluorescence dye. Another, the only equipment required is a simple centrifuge and a isothermal amplification equipment in the LAMP assay. Therefore, this technique performs multiplex pathogen can detection for thirteen different respiratory pathogens at once with 1 hour time consumption, while conventional culture methods are tiresome and typically require up to 72 hours. Consequently, it is worth to emphasize the advantages offered by the LAMP assay and that the protocol can be viewed as a simple and powerful approach for detecting pathogens in the absence of sophisticated equipment.

Our study has several limitations. First, the number of samples in this study was relatively small. Therefore, these results need to be verified in a larger sample size. Second, respiratory pathogens were identified by LAMP assay in the study while the results were not parallelly investigated by PCR due to resource problems. More detailed studies are needed to clarify the difference in detection performance between the 2 methods.

## 5 Conclusions

In summary, the clinical performance of the LAMP method is reliable and showing superior simplicity, sensitivity, specificity, and user-friendly handling compared with traditional bacteria culture method. It is a useful and cost-effective method, which have a good potential to be implemented for diagnosing pathogens in primary labs without any need for expensive equipment or specialized techniques in resource-limited areas of China. Finally, a large-scale population verification for the performance of LAMP assay will facilitate clinical application of this technology and that will be the focus of our next work.

## Acknowledgments

The author would like to thank other colleagues whom were not listed in the authorship of Center for Cardiovascular Diseases, Clinical Core Laboratory and Center for Precision Medicine, Meizhou People's Hospital (Huangtang Hospital), Meizhou Hospital Affiliated to Sun Yat-sen University for their helpful comments on the manuscript. This study was supported by National Key Research and Development Program of China (Grant No.: 2016YFD0500405 to Dr Pingsen Zhao), National Key Research and Development Program of China (Grant No.: 2017YFD0501705 to Dr. Pingsen Zhao), Natural Science Foundation of Guangdong Province, China (Grant No.: 2016A030307031 to Dr. Pingsen Zhao), Medical Scientific Research Foundation of Guangdong Province, China (Grant No.: A2016306 to Dr Pingsen Zhao) and Key Scientific and Technological Project of Meizhou People's Hospital (Huangtang Hospital), Meizhou Hospital Affiliated to Sun Yat-sen University, Guangdong Province, China (Grant No.: MPHKSTP-20170102 to Dr Pingsen Zhao), Medical Scientific Research Foundation of Guangdong Province, China (Grant No.: A2017404 to Dr Jingyuan Hou).

## Author contributions

Pingsen Zhao conceived and designed the experiments; Jingyuan Hou and Pingsen Zhao recruited subjects and collected clinical data. Heming Wu, Xing Zeng and Hui Rao conducted the laboratory testing. Pingsen Zhao and Jingyuan Hou prepared the manuscript.

**Conceptualization:** Pingsen Zhao.

**Data curation:** Jingyuan Hou, Heming Wu, Hui Rao, Pingsen Zhao.

**Formal analysis:** Jingyuan Hou, Heming Wu, Xing Zeng, Pingsen Zhao.

**Funding acquisition:** Pingsen Zhao.

**Investigation:** Pingsen Zhao.

**Methodology:** Jingyuan Hou, Heming Wu, Xing Zeng, Hui Rao, Pingsen Zhao.

**Project administration:** Pingsen Zhao.

**Resources:** Jingyuan Hou, Pingsen Zhao.

**Software:** Jingyuan Hou, Heming Wu, Xing Zeng, Hui Rao, Pingsen Zhao.

**Supervision:** Pingsen Zhao.

**Validation:** Jingyuan Hou, Heming Wu, Xing Zeng, Hui Rao, Pingsen Zhao.

**Visualization:** Pingsen Zhao.

**Writing – original draft:** Jingyuan Hou, Pingsen Zhao.

**Writing – review & editing:** Pingsen Zhao.
